# The effect of therapeutic plasma exchange on the inflammatory response in septic shock: a secondary analysis of the EXCHANGE-1 trial

**DOI:** 10.1186/s40635-025-00725-z

**Published:** 2025-02-14

**Authors:** Andrea Sauer, Klaus Stahl, Benjamin Seeliger, Pedro David Wendel-Garcia, Felix Lehmann, Julius J. Schmidt, Bernhard M. W. Schmidt, Tobias Welte, Konrad Peukert, Lennart Wild, Christian Putensen, Sascha David, Christian Bode, for the BonHanZA Study Group, Eva-Maria Kleinert, Rolf Erlebach, Rea Andermatt, Daniel Andrea Hofmaenner, Mattia Mueller, Reto Schuepbach, Alix Buhlmann, Thorben Pape, Ann-Kathrin Rath, Bahar Nalbant, Jannik Ruwisch, Caroline Feuerborn, Philippe Kruse

**Affiliations:** 1 Department of Anesthesiology and Intensive Care Medicine, University Hospital Bonn, Bonn, Germany; 2 Department of Gastroenterology, Hepatology, Infectious Diseases and Endocrinology, Hannover Medical School, Hannover, Germany; 3 Department of Respiratory Medicine, Hannover Medical School, Hannover, Germany; 4 Biomedical Research in End-Stage and Obstructive Lung Disease (BREATH), Hannover Medical School (MHH), German Center for Lung Research (DZL), Hannover, Germany; 5 Institute of Intensive Care Medicine, University Hospital Zurich, Rämistrasse 100, 8091 Zurich, Switzerland; 6 Department of Nephrology and Hypertension, Hannover Medical School, Hannover, Germany

**Keywords:** Extracorporeal treatment, Plasmapheresis, Immune response, Damage-associated molecular pattern molecules, Cell-free nucleic acids, Blood purification, Fresh frozen plasma, Sepsis, Precision medicine

## Abstract

**Background:**

Sepsis and septic shock, defined by a profound immune dysregulation, are among the leading causes of death in the intensive care unit (ICU). Despite advances in understanding the underlying pathophysiology, evidence for specific immunomodulatory treatment does not exist to date. Therapeutic plasma exchange (TPE) represents an adjunctive treatment approach to rebalance immune homeostasis. In the EXCHANGE-1 trial, we recently demonstrated a rapid hemodynamic improvement, possibly caused by the removal of harmful mediators and the replacement of protective plasma proteins. The aim of this secondary analysis is to further characterize the underlying immunomodulatory effects and to identify biomarkers that may predict treatment response.

**Methods:**

This secondary analysis included patients in early septic shock (< 24 h duration) and a norepinephrine (NE) dose of ≥ 0.4 μg/kg/min. Patients were randomized 1:1 to receive standard of care (SOC) or SOC + one single TPE and plasma samples were collected before and after TPE. Within-group and between group effects of circulating levels of acute-phase proteins [CRP and Pentraxin3 (PTX3)], inflammatory mediators (IL-4, IL-6, IL-8, IL-10, TNF-α, IL-2Rα/CD25) and damage-associated molecular pattern (DAMP) [cell-free DNA (cfDNA)] were analyzed via paired *t* test or Wilcoxon signed-rank test and a mixed-effects model. Multivariate mixed‐effects modeling of NE and lactate reduction was performed to investigate if cfDNA could be associated with treatment response to TPE.

**Results:**

TPE led to a significant reduction in circulating acute-phase protein levels (CRP *p* = 0.00976, PTX3 *p* = 0.0001). Pro-inflammatory cytokines, such as circulating TNF-α-, IL-6- und IL-8-levels, were significantly reduced in both groups with no significant difference between treatment groups except for IL-2Rα/CD25 (*p* ≤ 0.0001). In a multivariate mixed-effects model, rising cfDNA levels over the first 6 h indicated refractoriness to SOC treatment regarding NE (*p* = 0.004) and lactate (*p* = 0.001), whereas those receiving TPE demonstrated sustained reductions in both parameters.

**Conclusions:**

In this secondary analysis of the EXCHANGE-1 trial adjunctive TPE is associated with the reduction of acute-phase proteins and IL-2Rα/CD25, however not with the reduction of pro-inflammatory cytokines. This phenomenon could contribute to the observed enhancement in hemodynamics among patients with septic shock. Furthermore, TPE may be particularly beneficial for patients with septic shock who exhibit rising levels of cfDNA.

**Supplementary Information:**

The online version contains supplementary material available at 10.1186/s40635-025-00725-z.

## Background

Sepsis, characterized as a dysregulated immune response to infection causing life-threatening organ dysfunction, remains a major healthcare burden [1]. As one of the leading causes of death with 11 million estimated deaths worldwide, sepsis accounts for 20% of all deaths globally [2]. Even though our knowledge of the underlying pathophysiology has grown tremendously over the last decades, there is still an unmet need for a targeted therapy against the hostʼs injurious response to infection. Therefore, therapeutic management of sepsis so far solely relies on source control, the administration of broad-spectrum antibiotics and supportive care including organ support [3, 4].

Septic shock, defined by hyperlactatemia and persisting hypotension, manifests as concurrent immunosuppression and hyperinflammation driven by inflammatory mediators, endothelial dysfunction, glycocalyx degradation, and a dysregulation of the coagulation and complement systems [4]. Neutrophil activation and cellular damage may cause the release of numerous cytokines, inflammatory mediators and endogenous molecules called damage-associated molecular patterns (DAMPs) leading to further deterioration fueled by the stimulation of pattern recognition receptors (PRRs) [3, 4]. However, hundreds of trials assessing strategies to modify the body’s immune response by mainly targeting single‐mediator molecules or single pathways have failed to demonstrate a clinical benefit. As sepsis is a dynamic, heterogeneous syndrome caused by the imbalances of several systems, the current challenge for future sepsis research lies not only in personalizing medicine but also in identifying novel multimodal treatment approaches.

The basic concept of therapeutic plasma exchange (TPE) in sepsis is to replace already depleted protective plasma proteins such as protein C and to simultaneously remove harmful circulating molecules that directly contribute to the development of the disease [5–7]. Theoretically, the removal of inflammatory mediators such as IL-6 and DAMPs (e.g. cfDNA) are biologically plausible contributors to the recently observed beneficial effects of TPE.

Our group has demonstrated that a TPE against fresh frozen plasma (FFP) from healthy donors as an adjunctive treatment in early (onset < 24 h) and severe (norepinephrine requirement ≥ 0.4 µg/kg/min) septic shock has led to a rapid hemodynamic stabilization, improvement of fluid balances, the elimination of damaging endothelial mediators such as von Willebrand factor (vWF), angiopoietin-2, soluble Tie2 (sTie-2), and the replenishment of protective factors including protein C, antithrombin III (AT-III) and a disintegrin and metalloproteinase with a thrombospondin type 1 motif, member 13 (ADAMTS13) [8, 9]. Several meta-analysis have suggested a potential survival benefit which has led to the development of two large RCTs which are about to start in Europe (EXCHANGE-2, NCT05726825) and Canada (PLEXSIS, NCT05093075) [10–12]. However, the effect of TPE on the humoral immune response in septic shock remains rather unclear [13].

This post‐hoc secondary analysis of the EXCHANGE-1 trial investigated if TPE modulates the dysregulated host response in septic shock. Therefore, we sought to further characterize the immune response of TPE in septic shock patients receiving adjunctive TPE compared to control patients treated by standard medical therapy (SOC) only. A diverse selection of biomarkers including acute‐phase proteins [CRP and Pentraxin-3(PTX3)], inflammatory mediators (TNF-α, IL-6, IL-8, IL-4, IL-10, IL-2Rα/CD25) as well as DAMPs (cfDNA), which are associated with an unfavorable prognosis and depict signaling pathways as potential therapeutic targets in sepsis, were investigated [14–22]. Furthermore, we sought to develop a biomarker-based approach to identify treatment responders for future clinical trials.

## Methods

### Study population

In this post‐hoc secondary analysis from a prospective, open label RCT (and its extension) (EXCHANGE-1 trial, clinicaltrials.gov Identifier: NCT04231994) trial data and blood samples were both obtained from patients with early and severe septic shock (onset < 24 h) requiring an NE dose of ≥ 0.4 µg/kg/min [8]. Patients were treated according to the “Surviving Sepsis Campaign Guidelines for Management of Sepsis and Septic shock: 2016” [1].

The trial was conducted according to the principles of the Declaration of Helsinki and was approved by the Institutional Review Board of the Hannover Medical School (No. 2786-2015 and No. 8852_MPG_23b_2020) and the University Hospital Bonn (No. 024/20). Written informed consent was provided by participants or their legal representatives prior to enrollment. The study was registered at clincaltrials.gov (Identifier: NCT04231994).

### Inclusion and exclusion criteria

Eligible patients were adults admitted to the intensive care unit (ICU) with early septic shock (onset of vasopressor use < 24 h prior to screening), and an NE requirement of ≥ 0.4 µg/kg/min despite adequate intravenous fluid resuscitation (≥ 30 ml/kg bodyweight crystalloids). Exclusion criteria included age < 18 years, pregnancy or breast feeding, end-stage chronic kidney disease, and presence of a directive to withhold life-sustaining treatment.

### Randomization, intervention

Randomization was concealed via sealed opaque envelopes. Patients were assigned in a 1:1 ratio to SOC or SOC in addition to one single session of TPE against FFP. TPE had to be performed within 6 h following randomization. Vascular access was established by central venous insertion of an 11-French two-lumen hemodialysis catheter. Based on previous experiences only a single TPE session was performed, since hemodynamic improvements were only achieved by the very first exchange [23]. TPE was performed against FFP, exchanging a fixed dose of 12 units of pooled donor plasma. Individual patient’s plasma volume was calculated in retrospect by a formula using patient weight and hematocrit [24]. In patients with acute kidney injury (AKI), renal replacement therapy (RRT) was interrupted for the duration of TPE. Blood samples were drawn at randomization and 6 h following randomization. NE dose was titrated every 10–15 min to maintain a mean arterial pressure (MAP) above 65 mmHg.

### Blood sample collection and measurement of parameters

Patient plasma samples were directly centrifuged at 2500 G for 10 min at room temperature after collection and plasma was stored in aliquots at − 80 °C until further processing. Levels of IL-4, IL-6, IL-8, IL-10, TNF-α, IL-2Rα/CD25, CRP and PTX3 were analyzed by multiplex immunoassay (Luminex Assay, Bio-Techne, Minneapolis, MN, USA) while Qubit™ double‐stranded (ds) DNA high‐sensitivity (hs) assay was used to measure cfDNA levels. NE dose and lactate levels were collected at baseline and after 6, 12 and 24 h of randomization.

### Statistical analysis

Statistical analysis was conducted with GraphPad Prism Software (Version 9.0, La Jolla, California) and the R environment for statistical computing version 4.1.2 (R Foundation for Statistical Computing, Vienna, Austria). Normality of data distribution was assessed prior with the D’Agostino-Pearson omnibus normality test and the Shapiro–Wilk normality test. Within-group effects between the chosen two time points (randomization, 6 h after randomization) were analyzed via paired *t* test or Wilcoxon signed-rank test as appropriate. Comparisons between groups were analyzed via a mixed-effects model. Inflammatory mediators were individually modeled in a mixed-effects model framework in which they were entered as dependent variables to evaluate the temporal effect of TPE on inflammatory mediators. Time and TPE/SOC on the other hand were entered as individual fixed effects terms, acknowledging their interaction and introducing patients as random effects. The reported *p* values refer to the interaction term of this model. To achieve normal distribution and for better comparability, biomarker data were log-transformed.

Patient characteristics at inclusion were compared by *t* test, Fisher’s exact test or the Kruskal–Wallis test as appropriate. Modeling of the temporal effect of TPE was approached by means of a linear mixed-effects model. NE and lactate were entered as dependent variables into the model, whereas the intervention (TPE/ SOC), delta cfDNA and time were entered as independent fixed effects including their interaction. Finally, per patient random intercepts were entered into the model. *p* values for individual fixed effects were obtained by Satterthwaite’s degrees of freedom method. Model fit was assessed using a likelihood ratio test of the full model with the effects in question against a “null model”. For all statistical analyses a two-tailed *p* value < 0.05 was considered statistically significant, with no correction for multiple testing being employed due to the exploratory character of this work.

## Results

### Patients

**Fig. 1:**
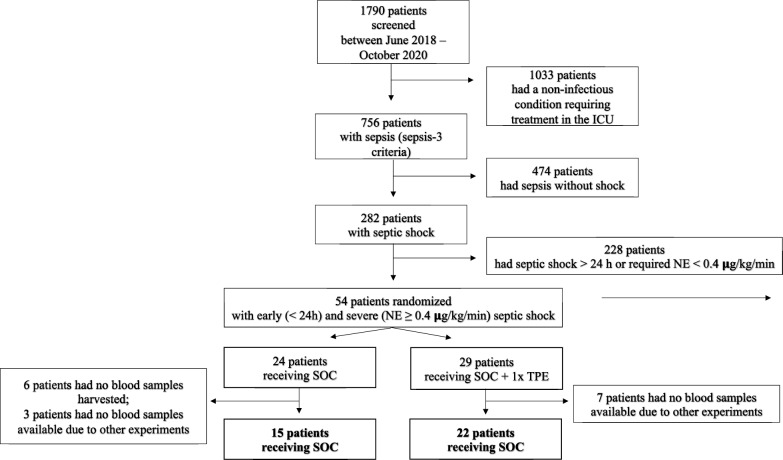
Flow chart of the study participants. Shown are screening, inclusion and randomization of patients. Inclusion criteria were early (< 24 h) and severe (noradrenaline (NE) dose ≥ 0.4 μg/kg/min despite adequate fluid resuscitation) septic shock. The study compared standard of care (SOC) with SOC + a single therapeutic plasma exchange (TPE) performed immediately after a 1:1 randomization

Of 1790 patients screened for sepsis between June 2018 and February 2021, 282 patients had septic shock according to the Sepsis-3 definition (Fig. 1). 54 patients with early and severe septic shock (onset < 24 h) requiring an NE dose of ≥ 0.4 µg/kg/min were recruited. Following randomization, 29 patients were assigned to the intervention group (SOC in addition to one single session of TPE) and 25 patients to the control group (SOC). Out of 54 participants, only 37 participants were analyzed because blood samples had not been drawn or had been used for other experiments in the remaining patients (Fig. 1).

### Cohort characterization

Demographics and clinical parameters were similar between groups at randomization (Tables 1 and 2). 76% of the patients were male with a median age of 55 years. Hypertension, obesity and diabetes mellitus were the most common comorbidities. Regarding clinical parameters (Table 2), the most common sites of infection were the lungs and abdomen, while the causative pathogen were predominantly gram+ and gram− bacteria. The median SOFA score at recruitment was 16, highlighting the significant burden of organ failure in these patients. This is further evidenced by the median vasopressor dose of 0.7 μg/kg/min, the high percentage of mechanically ventilated patients (89%), and the substantial proportion of patients receiving RRT (59%).

**Table 1:** Baseline demographics at study inclusion

Category	All *n* = 37	SOC *n* = 15	TPE *n* = 22
Age in years (median, [IQR])	53.00 [49.00, 60.00]	52.00 [46.50, 62.50]	53.50 [50, 59.75]
Sex			
Male	27 (73.0)	12 (80.0)	15 (68.2)
Female	10 (27.0)	3 (20.0)	7 (31.8)
BMI kg/m^2^ (median, [IQR])	25.40 [22.49, 31.14]	25.42 [23.48, 34.86]	24.89 [21.52, 31.13]
Comorbidities			
Obesity	12 (32.4)	5 (33.3)	7 (31.8)
Hypertension	12 (32.4)	3 (20.0)	9 (40.9)
Diabetes	7 (18.9)	4 (26.7)	3 (13.6)
COPD	3 (8.1)	1 (6.7)	2 (9.1)
CHF	5 (13.5)	2 (13.3)	3 (13.6)
CAD	1 (2.7)	0 (0.0)	1 (4.5)
CKD	7 (18.9)	3 (20.0)	4 (18.2)
Immunosuppression	9 (24.3)	3 (20.0)	6 (27.3)
SOT or HSCT	5 (13.5)	3 (20.0)	2 (9.1)

Shown are demographic characteristics at randomization for patients receiving standard of care treatment (SOC) as well as patients receiving additive therapeutic plasma exchange (TPE)

*BMI* body mass index, *CAD* coronary artery disease, *CHF* congestive heart failure, *CKD* chronic kidney disease, *CNS* central nervous system, *COPD* chronic obstructive pulmonary disease, *HSCT* hematopoietic stem cell transplant, *SOT* solid organ transplant

**Table 2:** Clinical parameters at study inclusion

Category	All *n* = 37	SOC *n* = 15	TPE *n* = 22
Sepsis onset—*n* (%)			
Ambulatory	22 (59.5)	9 (60.0)	13 (59.1)
Hospital	15 (40.5)	6 (40.0)	9 (40.9)
Site of infection—*n* (%)			
Pulmonary	22 (59.5)	9 (60.0)	13 (59.1)
Abdominal	10 (27.0)	4 (26.7)	6 (27.3)
Soft tissue	2 (5.4)	2 (13.3)	0 (0.0)
Endocarditis	1 (2.7)	0 (0.0)	1 (4.5)
CNS	1 (2.7)	0 (0.0)	1 (4.5)
Mixed	0 (0.0)	0 (0.0)	0 (0.0)
Identified pathogen—*n* (%)			
Gram+	9 (24.3)	4 (26.7)	5 (22.7)
Gram−	11 (29.7)	4 (26.7)	7 (31.8)
Fungal	3 (8.1)	1 (6.7)	2 (9.1)
Viral	3 (8.1)	2 (13.3)	1 (4.5)
Mixed	2 (5.4)	0 (0.0)	2 (9.1)
Non-identified	9 (24.3)	4 (26.7)	5 (22.7)
SOFA score [points, mean (SD)]	16.22 (2.66)	16.40 (3.07)	16.09 (2.41)
Norepinephrine dose (µg/kg/min, median [IQR])	0.58 [0.46, 0.82]	0.49 [0.45, 0.64]	0.64 [0.49, 0.87]
Mechanical ventilation *n* (%)	34 (91.9)	13 (86.7)	21 (95.5)
Oxygenation index (PaO_2_/FiO_2_) (median [IQR])	130.00 [95.00, 186.00]	165.00 [119.00, 225.50]	122.50 [95.92, 167.50]
ECMO—*n* (%)			
vv-ECMO	9 (24.3)	4 (26.7)	5 (22.7)
va-ECMO	2 (5.4)	2 (13.3)	0 (0.0)
Renal replacement therapy—*n* (%)	22 (59.5)	8 (53.3)	14 (63.6)
Lactate (median [IQR])	3.90 [2.68, 5.90]	3.90 [3.30, 5.75]	3.80 [2.54, 5.88]
Organ failure—*n* (%)			
Respiration	36 (97.3)	14 (93.3)	22 (100.0)
Coagulation	19 (51.4)	7 (46.7)	12 (54.5)
Liver	18 (48.6)	8 (53.3)	10 (45.5)
Cardiovascular	37 (100.0)	15 (100.0)	22 (100.0)
CNS	36 (97.3)	15 (100.0)	21 (95.5)
Renal	28 (75.7)	11 (73.3)	17 (77.3)
Leukocytes in G/l (median [IQR])	16.60 [7.93, 19.80]	16.80 [7.35, 19.50]	13.73 [8.17, 20.60]
Mortality	20 (54.1)	7 (46.7)	13 (59.1)

Shown are clinical characteristics at randomization for patients receiving standard of care treatment (SOC) as well as patients receiving additive therapeutic plasma exchange (TPE)

*CNS* central nervous system, *CRP* C-reactive protein, *ECMO* extracorporeal membrane oxygenation (*vv* venovenous, *va* venoarterial), *SOFA* Sequential organ failure assessment

### Effect of TPE on acute‐phase proteins

**Fig. 2:**
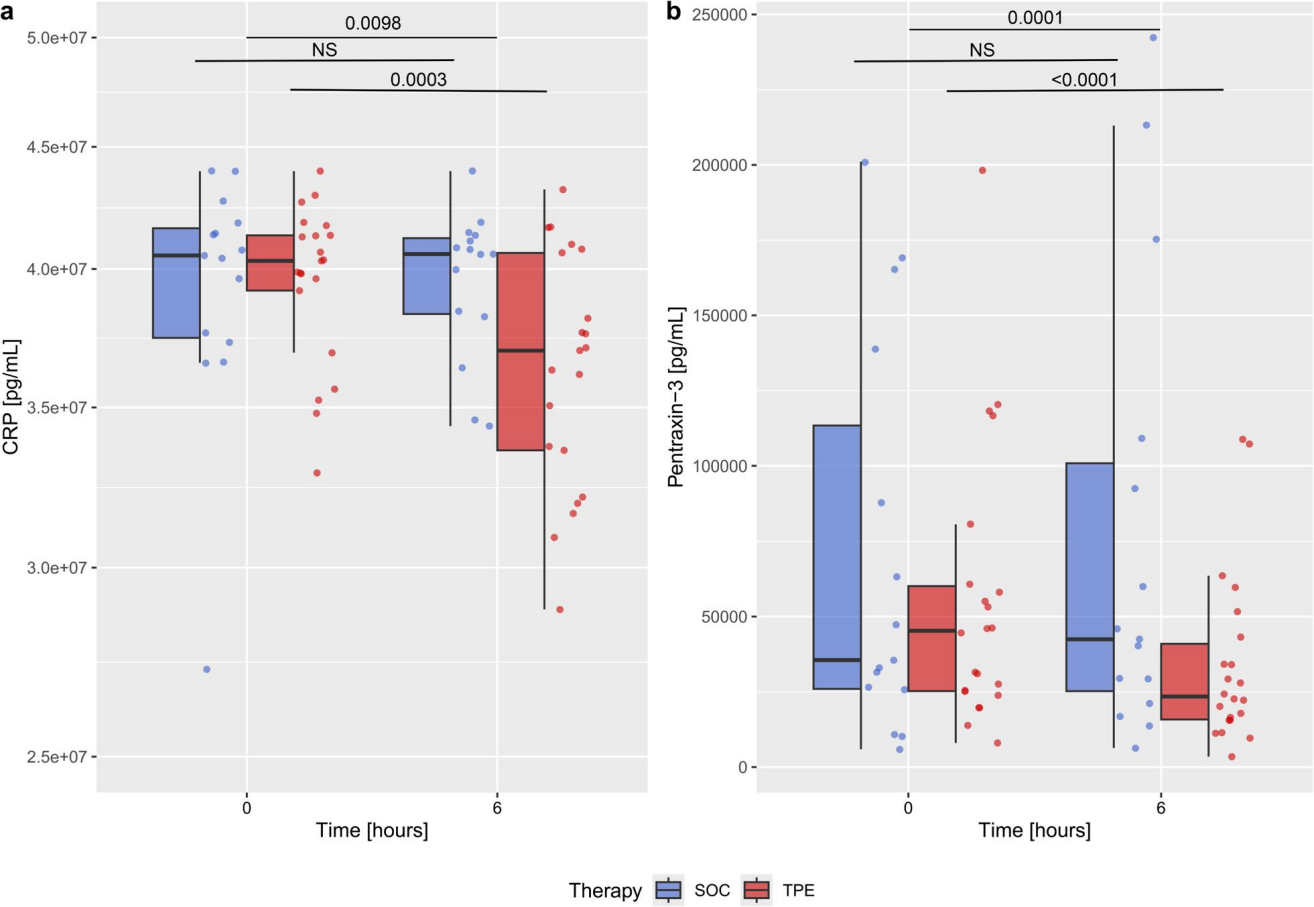
Effect of therapeutic plasma exchange on CRP and PTX3 levels in early septic shock. Box and whisker plots of CRP (**a**) and PTX3 (**b**) serum concentrations at randomization and 6 h after in patients with septic shock who received either standard of care (SOC) alone or SOC in combination with therapeutic plasma exchange (TPE)

Concentrations of the acute‐phase protein CRP remained high in the SOC group but significantly decreased in the TPE group compared to the time of randomization (*p* = 0.0003, Fig. 2a). Similar results were obtained for PTX3 (Fig. 2b, p ≤ 0.0001). A mixed-effects model was applied to reveal between group differences over time. Levels of CRP and PTX3 significantly decreased in the TPE group compared to the SOC group (*p* = 0.00976; *p* = 0.0001, respectively).

### Effect of TPE on inflammatory cytokines

**Fig. 3:**
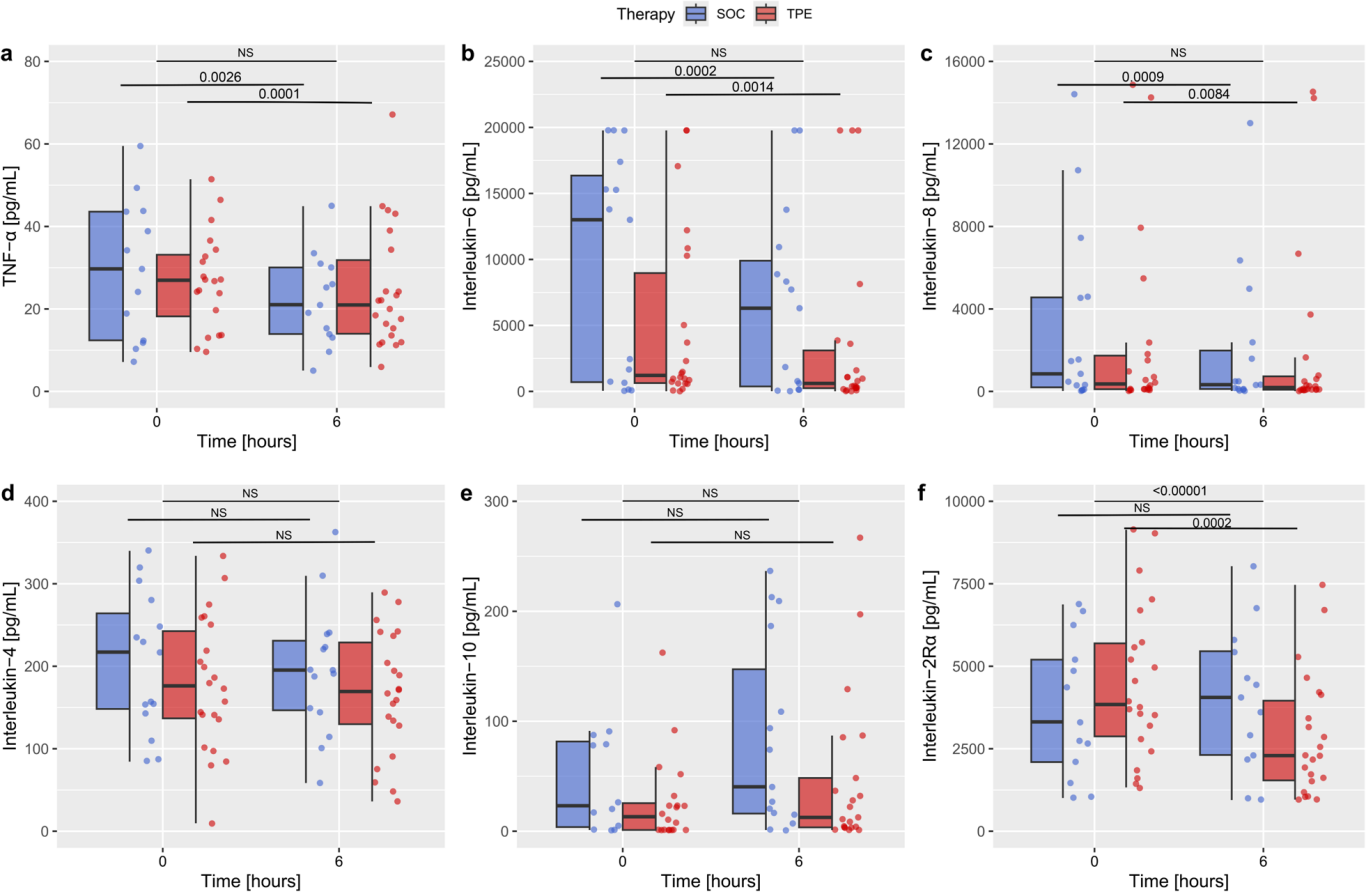
Effect of therapeutic plasma exchange on inflammatory mediator levels in patients with septic shock. Box and whisker plots of proinflammatory cytokines TNF-α (**a**), IL-6 (**b**), IL-8 (**c**), anti-inflammatory cytokines IL-4 (**d**), IL-10 (**e**) and inflammatory mediator IL-2Rα/CD25 (**f**) serum concentrations at randomization and 6 h after in patients with septic shock who received either standard of care (SOC) alone or SOC in combination with therapeutic plasma exchange (TPE)

Concentrations of circulating proinflammatory cytokines TNF-α, IL-6 and IL-8 were significantly reduced in the TPE group (Fig. 3a–c; *p* = 0.0001, *p* = 0.0014 and *p* = 0.0009, respectively). Patients that received only SOC also demonstrated a significant reduction in the measured cytokines (Fig. 3a–c; TNF-α: *p* = 0.0026, IL-6: *p* = 0.0002 and IL-8: *p* = 0.0084). A mixed-effects model showed no significant difference between groups over time.

Circulating concentrations of anti-inflammatory cytokines IL-4 and IL-10 were unchanged in both groups (Fig. 3d–e). In contrast, circulating concentration of IL-2Rα/CD25, a contributor to immunosuppression [25], plateaued 6 h after randomization in the SOC group while a statistically significant decrease was observed in the TPE group (Fig. 3e, p = 0.0002). A significant difference in IL-2Rα/CD25 levels between groups could be recorded over time (*p* ≤ 0.00001).

### Influence of cfDNA elevation on NE and lactate response

**Fig. 4:**
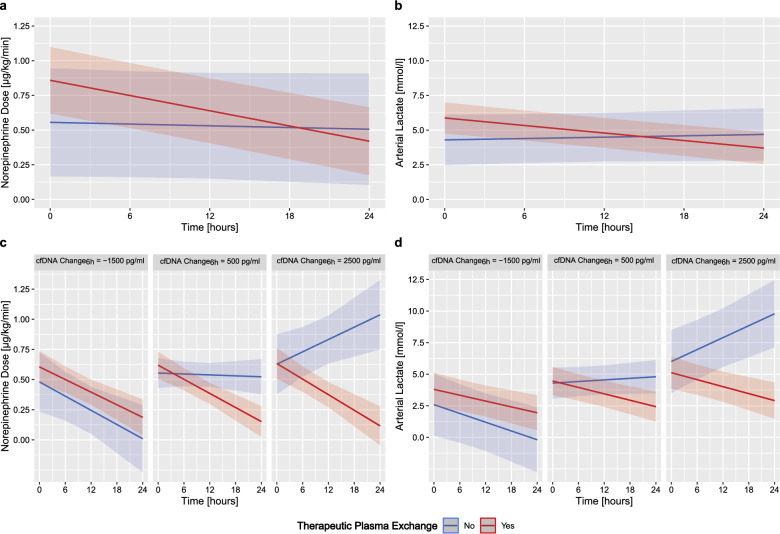
Predicted NE dose and lactate level response to therapeutic plasma exchange stratified by cfDNA change*.* The overall predicted response of **a** NE and **b** lactate over time to TPE is presented. Furthermore, the mediating effect of cfDNA changes over the initial 6 h on **c** NE and **d** lactate response are presented. Estimated values were calculated using a triple interaction model with TPE/SOC and time, as well as all simple interactions terms between fixed effects. The model indicates that SOC patients with increasing delta cfDNA levels experienced diminishing NE dose and lactate level reductions over 24 h in contrast to patients under TPE which experienced sustained NE reductions across all levels of cfDNA (NE: *p* = 0.004, lactate: *p* = 0.001). The thresholds for cfDNA changes represent the mean ± one standard deviation. Solid lines represent predicted mean effects with their 95% confidence interval depicted as shaded area

Our findings show that overall patients receiving TPE experienced a reduction in NE and lactate levels over the initial 24 h as opposed to the SOC group (Fig. 4a and b). Although statistically not significant, there was a trend toward a decrease in cfDNA concentration in the TPE group (Supplementary Table 1). We analyzed baseline cfDNA levels and changes in cfDNA as biomarkers to assess their impact on NE and lactate response (Supplementary Tables 2 to 4). Our findings indicate that baseline cfDNA did not predict therapeutic outcomes. However, in the SOC group, patients with rising cfDNA from randomization to 6 h showed an increase in NE dose over the initial 24 h (Fig. 4c). In contrast, patients receiving TPE had sustained NE reductions, regardless of cfDNA elevation. Specifically, with cfDNA increases of 500 pg/ml, the NE dose reduction trajectories for TPE and SOC began to diverge. At cfDNA elevations above 2500 pg/ml, the SOC group required higher NE doses, while NE reductions continued in the TPE group (Fig. 4c). Similar patterns were observed for lactate levels (Fig. 4d): SOC patients exhibited slight lactate increases above cfDNA levels of 500 pg/ml and a marked increase at 2500 pg/ml, whereas lactate levels continued to decrease in the TPE group (Fig. 4d).

## Discussion

This post‐hoc secondary analysis of a prospective bicentric RCT examined the immunomodulatory effects of TPE in septic shock patients. In contrast to SOC, we found that TPE reduced the levels of proinflammatory acute‐phase proteins (CRP and PTX3) and immunosuppressive IL-2Rα/CD25 while there was no effect on inflammatory cytokine levels. In a multivariate mixed-effects model, rising cfDNA levels in patients indicated refractoriness to SOC treatment concerning NE and lactate, whereas those receiving TPE demonstrated sustained reductions in both parameters.

The acute-phase proteins CRP and PTX3 both correlate with increased mortality in septic shock and higher levels have been detected in non-survivors [17, 18, 26]. Short pentraxin CRP and long pentraxin PTX3 are not only sepsis biomarkers but have also been shown to induce the upregulation of cell adhesion molecules on immune cells and trigger complement activation [19]. This underlines that the removal of these biomarkers and concurrently disease mediators could be of potential significance.

Notably, circulating levels of proinflammatory cytokines TNF-α, IL-6 and IL-8 fell in both groups 6 h after randomization while there was no significant difference in circulating IL-4 and IL-10 levels. A rapid decrease of these proinflammatory cytokine levels has already been described in the literature, and in conjunction with our results, it is questionable whether their removal is responsible for the hemodynamic stabilization observed in our septic shock patients [27]. Observational uncontrolled trials investigating the effect of cytokine adsorption have repeatedly reported reduced cytokine levels after the appropriate intervention [28, 29]. Nevertheless, when controlled trials evaluated the removal of IL-6, as sort of a surrogate signature interleukin representative, it was never lower than the control group [30–32]. This is very much in line with our findings using TPE to remove circulating molecules. One interpretation could be that the highly dynamic cytokine response is way too fast for a successful intervention but that downstream pathways, such as acute phase proteins and DAMPs are more reasonable targets for a blood purification approach.

Circulating IL-2Rα/CD25 levels were decreased in the TPE group compared to the SOC group. Soluble IL-2Rα/CD25 can bind to IL-2, potentially acting as a decoy receptor that sequesters IL-2 and prevents it from interacting with cell surface IL-2 receptors. Removing soluble IL-2Rα/CD25 could affect the balance between pro-inflammatory and regulatory immune responses, potentially exacerbating or ameliorating disease symptoms depending on the context. Increased levels have been reported in patients with sepsis, cancer as well as autoimmune diseases and are prognostic for a poor outcome [20, 21]. Therefore, reduction of soluble IL-2Rα/CD25 by TPE could lead to increased IL-2 availability, enhanced T cell activation and proliferation, and potential changes in immune regulation and homeostasis.

Precision medicine approaches, including prognostic and predictive enrichment, have been applied in various fields to subgroup patients for clinical trials based on their biology [33–35]. Due to the heterogeneity of sepsis and study population predictive enrichment aims at validating a subgroup that will benefit from a specific therapy by identifying distinct markers [36]. Bhagela et al. recently stratified early sepsis patients into five distinct endotypes, which vary in disease severity and might respond differently to specific therapies [37]. Multivariate mixed‐effects modeling of NE and lactate reduction was performed to investigate the potential mediation effect of cfDNA on TPE treatment response. In a triple interaction term with TPE/SOC and time, baseline cfDNA did not emerge as a predictor of hemodynamic response to TPE. Yet, the model exhibits that SOC patients with increasing delta cfDNA levels experienced increasing NE dose and lactate levels over 24 h in contrast to patients under TPE, who experienced sustained NE reductions across all levels of cfDNA. High levels of cfDNA may primarily reflect the degree of disease severity and clinical deterioration [38, 39]. This may explain why TPE is more beneficial in this certain subset of patients. In fact, cfDNA is a promising prognostic and predictive biomarker of tissue injury and disease severity that has been linked to poor outcomes in septic patients [14–16, 40]. Dwivedi et al. confirmed in a retrospective study of 80 patients with severe sepsis that cfDNA can predict ICU mortality and surpass available severity scoring systems and other biomarkers including procalcitonin, IL-6, thrombin and protein C [41, 42]. Higher cfDNA levels at admission were observed in later non-survivors compared to survivors, emphasizing that higher levels correspond with disease severity and the degree of tissue injury [14]. Current findings may help to identify pathological pathways of therapy refraction in septic shock, and if the cfDNA dynamics can be predicted, aid in personalizing TPE use in septic shock.

Our study has several limitations. Most importantly, the small sample size, the bicentric setting and the unavailability of some of the blood samples from the cohort may influence the significance of the results including potential type I and II errors. Furthermore, our study investigated only a limited number of biomarkers that cannot fully depict the release kinetics of each single biomarker and the complexity of the dysregulated immune response seen in septic patients. Future studies should add additional biomarkers associated with endothelial damage, apoptosis, glycocalyx degradation, coagulation and complement system. Advanced immune cell function assessments possibly depicting distinct immune response patterns to TPE were also not performed. They could represent a fundamental part of assessing immunomodulatory activity.

Since TPE was performed within 6 h of randomization, TPE could have been performed very early or late in this timeframe potentially influencing biomarker levels. Repeated measurements at preselected timepoints including at the beginning and end of TPE and the days following treatment should be implemented in follow-up trials to capture the variability due to biomarker assessment time.

## Conclusions

The current study demonstrates that adjunctive TPE therapy in septic shock is not associated with the removal of proinflammatory cytokines but with the removal of inflammatory acute-phase proteins and immunosuppressive IL-2Rα/CD25, which may contribute to the rapid hemodynamic improvement recently observed in septic shock patients. In addition, TPE may be particularly beneficial for patients with septic shock who exhibit rising levels of cfDNA.

## Supplementary Information

Supplementary Material 1. Figure 1. Effect of therapeutic plasma exchange on cfDNA levels in early septic shock. Box and whisker plot of damage-associated molecular pattern (DAMP) cfDNA serum concentrations at randomization and 6h after in patients with septic shock who received either standard of care (SOC) alone or SOC in combination with therapeutic plasma exchange (TPE).

Supplementary Material 2.

## Data Availability

Study material and datasets are available from the corresponding author upon reasonable request.

## Abbreviations

ADAMTS13: A disintegrin and metalloprotease with thrombospondin-1-like domains
AKI: Acute kidney injury
AT-III: Antithrombin-III
BMI: Body mass index
CAD: Coronary artery disease
cfDNA: Cell-free DNA
CKD: Chronic kidney disease
CHF: Congestive heart failure
COPD: Chronic obstructive pulmonary disease
CRP: C-reactive protein
DAMP: Damage-associated molecular pattern
ECMO: Extracorporeal membrane oxygenation (vv = venovenous, va = venoarterial)
FFP: Fresh frozen plasma
HSCT: Hematopoietic stem cell transplant
ICU: Intensive care unit
IL: Interleukin
MAP: Mean arterial pressure
NE: Norepinephrine
PRR: Pattern recognition receptor
PTX3: Pentraxin3
RCT: Randomized controlled trial
RRT: Renal replacement therapy
SOC: Standard of care
SOFA: Sequential Organ Failure Assessment
SOT: Solid organ transplant
sTIE2: A soluble receptor of tyrosine kinase with immunoglobulin-like and EGF-like domains 2
TPE: Therapeutic plasma exchange
vWF: Ag: Von Willebrand factor antigen
