# Supplementary figures and images for: The effect of therapeutic plasma exchange on the inflammatory response in septic shock: a secondary analysis of the EXCHANGE-1 trial
Source: Intensive Care Med Exp. 2025 Feb 14;13:18. doi: 10.1186/s40635-025-00725-z (PMC11828778; doi:10.1186/s40635-025-00725-z)

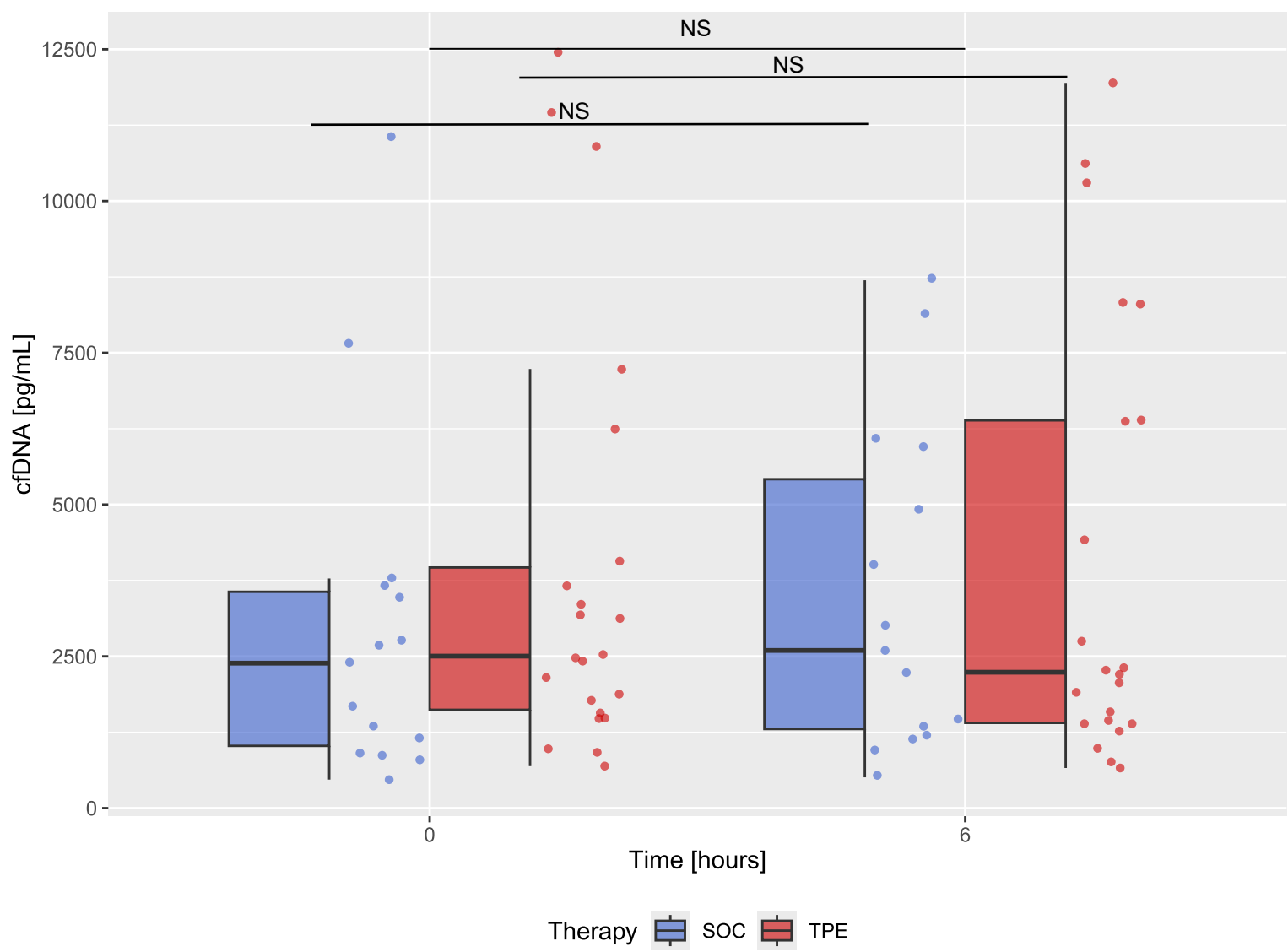

Supplement: Supplementary file 1 — Supplementary Material 1. Figure 1. Effect of therapeutic plasma exchange on cfDNA levels in early septic shock. Box and whisker plot of damage-associated molecular pattern (DAMP) cfDNA serum concentrations at randomization and 6h after in patients with septic shock who received either standard of care (SOC) alone or SOC in combination with therapeutic plasma exchange (TPE). [file 40635_2025_725_MOESM1_ESM.pdf]
