# Supplementary material for: The effect of therapeutic plasma exchange on the inflammatory response in septic shock: a secondary analysis of the EXCHANGE-1 trial
Source: Intensive Care Med Exp. 2025 Feb 14;13:18. doi: 10.1186/s40635-025-00725-z (PMC11828778; doi:10.1186/s40635-025-00725-z)
Supplement: Supplementary file 2 — Supplementary Material 2. [file 40635_2025_725_MOESM2_ESM.docx]

**Supplementary table 1: Linear mixed-effects model for predicted NE doses over time stratified by treatment**

|  | Estimates | 95% confidence interval | p |
| --- | --- | --- | --- |
| Intercept | 0.556 | 0.169 - 0.942 | **0.007** |
| TPE | 0.303 | -0.151 - 0.757 | 0.197 |
| Time [hours] | -0.002 | -0.011 - 0.007 | 0.654 |
| Interaction: TPE x Time [hours] | -0.016 | -0.027 - -0.006 | **0.003** |

**Supplementary table 2: Linear mixed-effects model for predicted lactate levels over time stratified by treatment**

|  | Estimates | 95% confidence interval | p |
| --- | --- | --- | --- |
| Intercept | 4.293 | 2.488 - 6.098 | **<0.0001** |
| TPE | 1.581 | -0.539 - 3.699 | 0.150 |
| Time [hours] | 0.016 | -0.034 - 0.067 | 0.525 |
| Interaction: TPE * Time [hours] | -0.107 | -0.166 -0.048 | **<0.001** |

**Supplementary table 3: Linear mixed-effects model for predicted NE doses over time stratified by treatment and cfDNA change**

|  | Estimates | 95% confidence interval | p |
| --- | --- | --- | --- |
| Intercept | 5.36E-01 | 4.09E-01 - 6.62E-01 | **<0.0001** |
| TPE | 7.98E-02 | -8.64E-02 - 2.46E-01 | 0.369 |
| cfDNA Change [pg/ml] | 3.69E-05 | -6.50E-05 - 1.39E-04 | 0.497 |
| Time | -5.87E-03 | -1.38E-02 - 2.18E-03 | 0.158 |
| Interaction: TPE * cfDNA Change [pg/ml] | -2.98E-05 | -1.37E-04 - 7.70E-05 | 0.600 |
| Interaction: TPE * Time [hours] | -1.31E-02 | -2.35E-02 - -2.81E-03 | **0.016** |
| Interaction: cfDNA Change [pg/ml] * Time [hours] | 9.17E-06 | 2.89E-06 - 1.54E-05 | **0.006** |
| Interaction: TPE * cfDNA Change [pg/ml] * Time [hours] | -1.02E-05 | -1.68E-05 - -3.49E-06 | **0.004** |

**Supplementary table 4: Linear mixed-effects model for predicted lactate levels over time stratified by treatment and cfDNA change**

|  | Estimates | 95% confidence interval | p |
| --- | --- | --- | --- |
| Intercept | 3.87E+00 | 2.60E+00 - 5.14E+00 | **<0.0001** |
| TPE | 4.25E-01 | -1.24E+00 - 2.09E+00 | 0.633 |
| cfDNA Change [pg/ml] | 8.54E-04 | -1.70E-04 - 1.88E-03 | 0.125 |
| Time | -1.33E-02 | -6.17E-02 - 3.50E-02 | 0.597 |
| Interaction: TPE * cfDNA Change [pg/ml] | -5.24E-04 | -1.60E-03 - 5.49E-04 | 0.364 |
| Interaction: TPE * Time [hours] | -6.95E-02 | -1.33E-01 - -5.93E-03 | **0.036** |
| Interaction: cfDNA Change [pg/ml] * Time [hours] | 6.83E-05 | 2.93E-05 - 1.07E-04 | **0.001** |
| Interaction: TPE * cfDNA Change [pg/ml] * Time [hours] | -7.20E-05 | -1.13E-04 - -3.02E-05 | **0.001** |
